# Assessing delays in accessing and completing radiotherapy for cervical cancer treatment: A multicenter survey of oncology providers in go further-funded countries in Sub-Saharan Africa

**DOI:** 10.1016/j.gore.2025.101810

**Published:** 2025-07-18

**Authors:** Caroline G. Kernell, Megan Kassick, Jessica M. George, Chidinma P. Anakwenze, Edward L. Trimble, Surbhi Grover

**Affiliations:** a UT Southwestern Medical School, 5323 Harry Hines Blvd, Dallas, TX 75390, United States of America; b University of Pennsylvania, Department of Radiation Oncology, Perelman Center for Advanced Medicine, 3400 Civic Center Blvd, Philadelphia, PA 19104, United States of America; c Botswana-UPenn Partnership, 1836 Princess Marina Hospital, Private Bag BO 320, Gaborone, Botswana; d Department of Population and Public Health Sciences Keck School of Medicine, University of Southern California, 2001 N Soto St, Los Angeles, CA 90032, United States of America; e MD Anderson Cancer Center, Department of Radiation Oncology, 1515 Holcombe Blvd, Houston, TX 77030, United States of America; f National Institutes of Health, National Cancer Institute, 9609 Medical Center Drive, Bethesda, MD 20892, United States of America

**Keywords:** Radiation oncology, Global health, Cervical cancer, Radiotherapy, Access to healthcare, Sub-Saharan countries

## Abstract

**Objective:**

Cervical cancer is a leading cause of cancer death in Sub-Saharan Africa. Go Further provides funds for prevention and screening in Sub-Saharan Africa, but access to treatment for invasive disease remains limited. This survey aims to assess delays in accessing curative-intent chemoradiotherapy for cervical cancer in countries receiving Go Further funding.

**Methods:**

Oncology providers in countries receiving Go Further funding (Botswana, Eswatini, Ethiopia, Kenya, Lesotho, Malawi, Mozambique, Namibia, Tanzania, Uganda, Zambia, Zimbabwe) and South Africa were invited to participate in a web-based survey beginning September 2023.

**Results:**

Fifteen oncology providers responded, with all countries except Malawi represented. Nearly half (46.3 %) reported wait time of ≥two months for chemoradiotherapy consultation. 93.3 %, 71.4 %, 53.7 % and 73.3 % had access to at least one CT, linear accelerator, cobalt, and brachytherapy machine, respectively. Majority (60 %) reported wait time of <one month to initiate chemoradiotherapy after staging. However, 28.6 % reported >95 % of patients complete external radiotherapy within 42 days, and 33 % reported >95 % of these patients receive brachytherapy. Only 26.7 % reported overall treatment time within 56 days for >95 % of patients. Lack of transportation, funding, and patient fear, were other treatment barriers reported.

**Conclusion:**

These results highlight the need for additional chemoradiotherapy resources in Go Further-funded countries. In addition to expanding radiotherapy, supplemental avenues to improve access in Sub-Saharan Africa include addressing barriers that increase wait times along the care continuum and implementing social support. This survey serves as a call to Go Further, highlighting the urgent need for resource allocation for cervical cancer treatment.

## Highlights

• Many cervical cancer treatment facilities in Sub-Saharan Africa lack adequate radiotherapy infrastructure.
• Delays in diagnosis and treatment initiation contribute to suboptimal cervical cancer outcomes.
• Key barriers to accessing care include transportation challenges, treatment fear, and financial hardship.
• Limited patient support services hinder adequate advanced-stage cervical cancer treatment.
• These findings highlight the urgent need to expand advanced-stage cervical cancer treatment efforts by Go Further.

## 1 Introduction

Cervical cancer is the fourth most commonly diagnosed cancer in women (Bray et al., 2018). Low- and middle-income countries are disproportionally affected, with Sub-Saharan Africa accounting for 80 % of those cases (Arbyn et al., 2020). Primary, secondary, and tertiary prevention strategies such as vaccination programs, screening programs, and treatment of precancerous lesions have decreased cervical cancer incidence and mortality rates in high-income countries, but screening programs in Sub-Saharan Africa are still developing (Arbyn et al., 2009). This region also faces a unique challenge: 67 % of the 38.4 million people with HIV globally live in Sub-Saharan Africa (Moyo et al., 2023). The risk of cervical cancer in women living with HIV increases 6-fold (Stelzle et al., 2021).

Launched in May 2018, Go Further is a public–private partnership between The United States President’s Emergency Plan for AIDS Relief, the George W. Bush Institute, the Joint United Nations Program on HIV/AIDS, Merck, and Roche aimed at combatting cervical cancer rates in Sub-Saharan Africa. Twelve countries are currently participating: Botswana, Eswatini, Lesotho, Malawi, Mozambique, Namibia, Zambia, Zimbabwe, Ethiopia, Kenya, Tanzania, and Uganda. Go Further investments target vaccination against Human Papilloma Virus (primary prevention) and cervical cancer screening (secondary prevention) (State USDo). By September 2021, Go Further screened 2.8 million women living with HIV for cervical cancer and treated 129,000 cases of pre-cancerous cervical lesions (State USDo, 2023).

Treating cases of invasive cervical cancer has not been a primary goal of Go Further. Patients in these countries that do present with invasive cervical cancer most commonly present with locally-advanced disease. Data from Botswana reported that, among patients with a known stage, over half presented with stage III or IV disease (Anakwenze et al., 2018).

Radiotherapy is an essential part of curative-intent treatment for locally-advanced cervical cancer. Despite the growing need, standard-of-care external beam radiotherapy is often unavailable as only 52 % and 39 % of African nations have access to external beam radiotherapy and brachytherapy, respectively (Elmore et al., 2021, Chargari et al., 2022, IAEA). Africa has a cervical cancer incidence rate over 2x higher than North America while having <20 % of North America’s radiotherapy capacity (Observatory, 2024, Cancer IAfRo, 2022). Brachytherapy, an essential component of cervical cancer treatment, is also difficult to access for those in Sub-Saharan Africa due to absence of machines and its requirement for unique resources including procedural expertise. Almost 50 % of the brachytherapy capacity of Africa is concentrated in only 2 countries: South Africa and Egypt (Agency, 2025).

We created a survey aiming to gather insight from oncology providers across Go Further-funded countries in Sub-Saharan Africa to characterize persistent delays in receiving curative-intent (radical) radiotherapy treatment for cervical cancer and identify areas in need of further funding for locally-advanced cervical cancer treatment. Given Go Further’s prominence in this area, this survey can aid in developing future funding initiatives by these organizations to further reduce barriers to cervical cancer screening and treatment in Sub-Saharan Africa.

## 2 Methods

A web-based survey was created by a multidisciplinary team including one medical student and two practicing radiation oncologists. Beginning in September 2023, an email invitation to participate in a web-based survey was sent to 19 oncology providers in countries receiving Go Further funding (Botswana, Eswatini, Ethiopia, Kenya, Lesotho, Malawi, Mozambique, Namibia, Tanzania, Uganda, Zambia, Zimbabwe). As the number of oncology providers in Sub-Saharan Africa is very low, the survey respondents were chosen through professional networks of the lead funding author and included 18 physicians (six medical, eight radiation, and four gynecologic oncologists) and one radiation therapist. In many instances, the respondent was the only practitioner of their specialty in the country. Monthly reminder emails were sent out to encourage survey completion. Responses were collected through Google Forms and summarized using descriptive statistics. Survey questions regarding machine availability and status detailed presence or absence of imaging machines, after which a drop-down response was populated if the imaging machine was present. Other survey questions included a “not applicable” option of not enough information was present to answer the question.

## 3 Results

### 3.1 Respondent characteristics

**Fig. 1:**
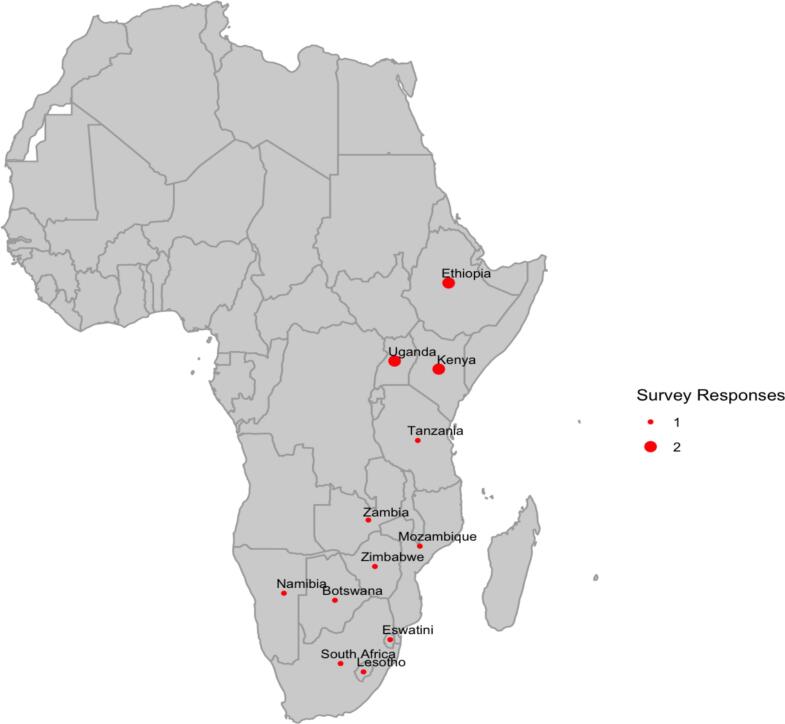
Distribution of survey responses across Sub-Saharan Africa.

Of the 19 invited oncology practitioners in South Africa and countries receiving Go Further funding, the response rate was 78.9 % (n = 15), of which 33.3 % (n = 5) were medical doctors, 46.7 % (n = 7) were radiation oncologists, 13.3 % (n = 2) were gynecologic oncologists, and 6.7 % (n = 1) were radiation therapists. Geographically, 69.2 % (9/13) target countries (Botswana, Eswatini, Lesotho, Mozambique, Namibia, South Africa, Tanzania, Zambia, and Zimbabwe) provided one survey response and 23.1 % (3/13) target countries provided 2 survey responses (Ethiopia, Kenya, and Uganda) (Fig. 1). Malawi was the only nation that did not have a representative respond to the survey. All but one respondent (93.3 %, n = 14) were employed at a public center, with 53.3 % (n = 8) reporting that their country had universal healthcare or another form of insurance that covers the costs associated with cancer diagnosis and chemoradiation.

### 3.2 Waiting times

When evaluating responses regarding wait times for patients from time of diagnosis to treatment consultation (n = 15), 40.0 % (n = 6) of respondents reported waiting times of <1 month (Table 1). For those who reported wait times >1 month, 26.7 % (n = 4) and 33.3 % (n = 5) indicated waiting times of <2 months and greater than 2 months, respectively. Regarding wait times for patients to receive a CT scan for staging (n = 15), 60.0 % (n = 9) of respondents reported wait times of less than 2 weeks, 13.3 % (n = 2) reported wait times of <1 month, and 6.7 % (n = 1) reported wait times >2 months. The distribution of responses regarding wait times for patients to receive a CT scan for simulation (n = 14) was: 35.7 % (n = 5) <2 weeks, 14.3 % (n = 2) <1 month, and 21.4 % (n = 3) >2 months. For wait times of patients to start curative-intent chemoradiation after staging (n = 15), majority of respondents reported wait times of <2 weeks (46.7 %, n = 7) or <1 month (20.0 %, n = 3). For wait times of patients to start brachytherapy after completion of external beam irradiation (n = 15), majority of respondents reported wait times of <2 weeks (46.7 %, n = 7) or <1 month (20.0 %, n = 3).

**Table 1:** Survey responses regarding wait times.

Question	Responses	
	N	%
What is the average waiting time for someone with newly diagnosed cervical cancer to be seen at the cancer treatment site in consultation?	15	
<1 month	6	40.0
<2 months	4	26.7
>2 months	5	33.3
What is the waiting time to get a CT scan for staging?	15	
<2 weeks	9	60.0
<1 month	2	13.3
<2 months	0	0.0
>2 months	1	6.7
Not applicable	3	20.0
What is the waiting time to get a CT scan for simulation?	14	
<2 weeks	5	35.7
<1 month	2	14.3
<2 months	0	0.0
>2 months	3	21.4
Not applicable	4	28.6
What is the waiting time to start curative-intent (radical) chemoradiation after staging?	15	
<2 weeks	7	46.7
<1 month	3	20.0
<2 months	1	6.7
>2 months	1	6.7
Not applicable	3	20.0
What is the waiting time to start brachytherapy after completion of external beam irradiation?	15	
<2 weeks	7	46.7
<1 month	3	20.0
<2 months	1	6.7
>2 months	1	6.7
Not applicable	3	20.0

### 3.3 CT scanner

All but one of the respondents (93.3 %, n = 14) reported having at least one CT machine in the facility (Table 2). Among those with at least one machine (n = 14), 71.4 % (n = 10) and 85.7 % (n = 12) of respondents used the machine for diagnosis and simulation, respectively. The most commonly reported number of machines available per site was 1 (35.7 %, n = 5) and 2 (42.9 %, n = 6). Siemens was the most commonly reported model of machine used (57.1 %, n = 8). Other models used included GE (21.4 %, n = 3), Toshiba (14.3 %, n = 2), and Phillips (7.1 %, n = 1). When asked if the machine was functional at present, 92.9 % (n = 13) of respondents reported “Yes”. The majority of respondents estimated that the average downtime per month occurred for <4 days (78.6 %, n = 11).

**Table 2:** Survey responses regarding machine availability and status.

Question	Responses	
	N	%
Does the treatment site have access to a CT scan?	15	
Yes	14	93.3
No	1	6.7
What is the purpose of the CT scanner? (check all that apply)	14	
Diagnostic Imaging	10	71.4
Radiation Simulations	12	85.7
How many CT scanners does the site have?	14	
1	5	35.7
2	6	42.9
3	2	14.3
4	1	7.1
What is the model and manufacturer of the CT scan?	14	
Siemens	8	57.1
Phillips	1	7.1
GE	3	21.4
Toshiba	2	14.3
Unknown	1	7.1
Is it functional at present?	14	
Yes	13	92.9
No	1	7.1
What is the average downtime per month?		
<4 days	11	78.6
<1 week	1	7.1
<2 weeks	1	7.1
>2 weeks	1	7.1
Does the treatment site have access to a linear accelerator?	14	
Yes	10	71.4
No	4	28.6
How many linear accelerators does the site have?	10	
1	5	50.0
2	1	10.0
3	3	30.0
4	1	10.0
What is the model and manufacturer of the machine?	10	
Varian	8	80
Seimens	1	10.0
Elekta	1	10.0
Unknown	1	10.0
Is it functional at present?	10	
Yes	8	80
No	2	20
What is the average downtime per month?	10	
<4 days	4	40.0
<1 week	4	40.0
<2 weeks	0	0.0
>2 weeks	2	20.0
Does the treatment site have access to a cobalt machine?	15	
Yes	8	53.3
No	7	46.7
How many cobalt machines does the site have?	8	
1	2	25.0
2	6	75.0
What is the model and manufacturer of the machine?	8	
Equinox	3	37.5
Bhabhatron	4	50.0
Terabalt	2	25.0
Theratrone	3	37.5
Unknown	0	0.0
Is it functional at present?	8	
Yes	4	50.0
No	4	50.0
What is the average downtime per month?	8	
<4 days	2	25.0
<1 week	2	25.0
<2 weeks	0	0.0
>2 weeks	4	50.0
Does the treatment site have access to a brachytherapy machine?	15	
Yes	11	73.3
No	4	26.7
How many brachytherapy machines does the site have?	11	
1	6	54.5
2	2	18.2
3	3	27.3
What is the model and manufacturer of the machine?	11	
Elekta	4	33.3
Seimens	1	8.3
Bebig	4	33.3
Equinox	1	8.3
Unknown	2	16.7
Is it functional at present?	11	
Yes	11	100.0
No	0	0.0
What is the average downtime per month?	11	
<4 days	10	90.9
<1 week	1	9.1
<2 weeks	0	0.0
>2 weeks	0	0.0

### 3.4 Linear accelerator

Among those who responded regarding linear accelerators (n = 14), 71.4 % (n = 10) reported having at least one linear accelerator in the facility (Table 2). For sites with at least one linear accelerator (n = 10), there were commonly 1 (50.0 %, n = 5) or 3 (30.0 %, n = 3) machines available per site. The most commonly reported model of linear accelerator was Varian at 80.0 % (n = 8). Varian Truebeam, CILNACix, 600C, Unique, Halcyon, and DMX were all represented. When asked if the machine was functional at present, 80.0 % (n = 8) reported “Yes”. The majority of respondents estimated that the average downtime per month occurred for less than 4 days (40.0 %, n = 4) or <1 week (40.0 %, n = 4).

### 3.5 Cobalt machine

Among respondents (n = 15), about half (53.3 %, n = 8) reported having at least one cobalt machine in the facility (Table 2). Most sites with at least one machine available (n = 8) reported having 2 machines available (75.0 %, n = 6). Bhabhatron was the most commonly reported model (50.0 %, n = 4), followed by Equinox (37.5 %, n = 3), Theratrone (37.5 %, n = 3), and Terabalt (25.0 %, n = 2). When asked if the machine was functional at present, 50.0 % (n = 4) of respondents reported “Yes”. Sites typically experienced an average downtime per month of greater than 2 weeks (50.0 %, n = 4).

### 3.6 Brachytherapy machine

Among respondents (n = 15), 73.3 % (n = 11) reported having at least one brachytherapy machine in the facility (Table 2). The majority of sites with at least one machine available (n = 11) reported having 1 (54.5 %, n = 6) or 3 (27.3 %, n = 3) machines available. Elekta and Bebig were the most commonly reported models at 33.3 % (n = 4) each. All respondents (n = 11) reported “Yes when asked if the machine was functional at present. The average downtime per month was rarely reported to be greater than 4 days (9.1 %, n = 1).

### 3.7 Treatment duration

Survey responses regarding treatment completion are summarized in Table 3. Distribution of responses estimating the proportion of patients who complete external beam irradiation within 42 days (n = 14) was fairly balanced: 21.4 % (n = 3) reported 0–49 %, 35.7 % (n = 5) reported 50–95 %, and 28.6 % (n = 4) reported >95 %. The majority of respondents reported that 50–95 % (40.0 %, n = 6) or greater than 95 % (33.3 %, n = 5) of patients who complete external beam irradiation complete brachytherapy. Among respondents (n = 15), 20.0 % (n = 3), 33.3 % (n = 5), and 26.7 % (n = 4) reported that 0–49 %, 50–95 %, and greater than 95 % of patients completed external beam irradiation and brachytherapy within 56 days, respectively.

**Table 3:** Survey responses regarding treatment completion.

Question		Responses	
		N	%
What proportion of patients who want treatment complete external beam irradiation within 42 days?		14	
0–49 %		3	21.4
50–95 %		5	35.7
>95 %		4	28.6
Not applicable		2	14.3
What proportion of patients who complete XRT get definitive brachytherapy?		14	
0–49 %		1	6.7
50–95 %		6	40.0
>95 %		5	33.3
Not applicable		3	20.0
What proportion of patients complete XRT and brachytherapy within 56 days?		15	
0–49 %		3	20.0
50–95 %		5	33.3
>95 %		4	26.7
Not applicable		3	20.0

### 3.8 Social support and communication

Survey responses regarding social support and communication are summarized in Table 4. Notably, most cancer facilities (73.3 %, n = 11) do not have resources to help patients with transportation, lodging, or meals during external beam irradiation and brachytherapy. All 15 respondents reported that their facility uses follow-up appointments to contact and monitor patients after treatment, and 46.7 % (n = 7) reported their facility also uses phone calls to follow-up with patients. Verbal communication by medical officers or physicians (80.0 %, n = 12) was the most commonly answered method to discuss the chemoradiation process and its side effects; additionally, cancer centers rely on verbal communication by nursing staff (60.0 %, n = 9), patient hand-outs (26.7 %, n = 4), and informational videos at the visit (6.7 %, n = 1). The most commonly reported barriers to treatment access were lack of transportation (86.7 %, n = 13), lack of funds (80.0 %, n = 12), and patient fear of treatment (73.3 %, n = 11). Regarding programs implemented to improve access to patient care (n = 14), patient navigation programs were most highly reported at 28.6 % (n = 4), followed by cancer awareness programs at 14.3 % (n = 2); free responses emphasized mass media/outreach programs, workforce training, using a multidisciplinary team model, and campaigns to increase patient volume.

**Table 4:** Survey responses regarding social support and communication.

Question	Responses	
	N	%
Does the cancer facility have resources to help patients with transportation, lodging, or meals during XRT and brachy?	15	
Yes	4	26.7
No	11	73.3
What programs has the cancer center implemented that have improved patient access to care? (Includes free responses)	14	
Navigation Program	4	28.6
Creating cancer awareness	2	14.3
Mass media/outreach programs	1	7.1
Direct appointment dates for RT at time of diagnosis within 2 weeks of initial exam	1	7.1
MDT with the gynae-oncology team with immediate referral of patients to oncology after they have been fully staged and discussed.	1	7.1
campaign to treat more patients over time and over weekends	1	7.1
Community Outreach	1	7.1
Private Wing (After working hours and weekends for paying patients) Hypofractionation protocols for taking more patients	1	7.1
Boarding for out-of-town patients	1	7.1
Workforce training	1	7.1
Multidisciplinary discussion of cases; Creation and use of Whatsapp group for guidance in terms of how to manage cases in different levels of health units.	1	7.1
What system is in place for the cancer center to contact patients who have undergone definitive chemoradiation for follow-up? (select all that apply)	15	
Follow up appointment/visit	15	100.0
Phone call	7	46.7
House visit	0	0.0
Online survey	0	0.0
Other	0	0.0
How does the cancer center communicate the process and/or side effects of chemoradiation treatment to patients? (select all that apply)	15	
Informational videos at clinic visit	1	6.7
Patient hand-outs	4	26.7
Verbal communication by nursing staff	9	60.0
Verbal communication by medical officers/physicians	12	80.0
What are the main barriers seen in the community to receiving chemoradiation treatment? (select all that apply)		
Lack of transportation	13	86.7
Lack of funds	12	80.0
Lack of knowledge about treatment	6	40.0
Lack of familial support	6	40.0
Patient fear of treatment	11	73.3
Social stigma	7	46.7
Seeking traditional medicine in lieu of treatment	9	60.0
Long Waiting time	1	6.7
Machine Breakdown	1	6.7

## 4 Discussion

This survey, completed by 15 oncology providers in Sub-Saharan Africa, provides insight into challenges in radiotherapy delays for cervical cancer treatment in the region. Limited diagnostic imaging and radiotherapy treatment machine availability, machine downtime contributing to longer overall treatment durations, and limited social support contribute to worse cancer outcomes for invasive cervical cancer in Sub-Saharan Africa. Though addressing treatment of invasive disease is not one of Go Further’s stated goals, the World Health Organization’s (WHO) *Global Strategy To Accelerate The Elimination Of Cervical Cancer As A Public Health Problem* highlights not only the need for HPV vaccination and cervical cancer screening, but also emphasizes expanding access to treatment of invasive disease (Organization, 2020). The WHO predicts that successful implementation of their proposed screening and treatment plan has the potential to save 300,000 women from death due to cervical cancer by 2030 (Organization, 2020). Addressing barriers that prevent timely access to screening and treatment and increase wait times along the care continuum, expanding the number of available and functioning radiotherapy machines, and implementing social support resources and communication methods aimed to reduce patient fear and increase knowledge, as illustrated in this survey, have potential to improve cervical cancer outcomes for patients in Sub-Saharan Africa. Although we did not cover the issue in our survey, we also acknowledge the importance of increasing access to universal health care covering cancer diagnosis and treatment. We know that many patients in Go Further countries experience high out-of-pocket expenses associated with cancer diagnosis and treatment.

### 4.1 Imaging needs

Access to imaging for both diagnosis and simulation for radiotherapy is limited in Sub-Saharan Africa. All but one respondent reported the presence of at least one CT scanner in their facility, though more than 75 % of respondents reported 2 or fewer machines available for the entire facility. Additionally, some facilities (14.2 %) reported average machine downtimes over 1 week per month, reducing imaging capacity by at least 25 % monthly. These factors limit patient throughput capacity, delaying access to timely cancer treatment. Within these facilities, functionality of CT scanners is also limited. Over one quarter and roughly 15 % of respondents indicated their CT scanner was not used for diagnostic imaging and radiation simulation, respectively. Increased access to imaging machines in Sub-Saharan Africa is essential, especially considering the wait times for receiving a CT scan for cancer staging and RT simulation. More than 25 % of respondents reported wait times greater than 2 months to receive staging or imaging, which may contribute to increased patient anxiety and worse cancer outcomes. These results highlight the dire need for increased funding for imaging machines with routine maintenance and are supported by other studies that similarly focus on access to imaging as a crucial area of improvement (Aderinto, 2023).

### 4.2 Wait times

In addition to wait times to receive a CT scan for diagnosis or simulation, patients with cervical cancer also face barriers in initiating curative chemoradiotherapy and brachytherapy. Although 40 % of respondents reported wait times <1 month between diagnosis and consultation for treatment evaluation, 33.3 % reported wait times longer than 2 months. With an estimated tumor volume doubling time of 302 days, these wait times delay treatment initiation and contribute to higher cervical cancer disease burden and poorer outcomes (Cosper et al., 2015). Delays were also seen in time to treatment initiation following initial consultation, and in brachytherapy initiation after completion of external beam irradiation. Less than half of respondents indicated wait times of 2 weeks or less for starting curative chemoradiation after staging and starting brachytherapy after completion of external beam radiotherapy. The importance of timely treatment for cervical cancer cannot be understated. Several studies have established that prolonged treatment time for combined external beam radiotherapy and brachytherapy past 56 days, or 8 weeks, is strongly associated with worse clinical outcomes (Fyles et al., 1992, Petereit et al., 1995). Since the establishment of concurrent chemoradiotherapy as standard-of-care for locally advanced cervical cancer in 1999 (Whitney et al., 1999); further investigations from the image guided intensity modulated external beam radiochemotherapy and MRI-based adaptive brachytherapy in locally advanced cervical cancer (EMBRACE) group have emerged providing suggestions for a limit of 49 days, or 7 weeks, for treatment combined external beam radiotherapy and brachytherapy (Pötter et al., 2021). Factors driving these long wait times are multifarious, influenced by the high cervical cancer disease burden in the region and lack of machine presence and functionality. Based on survey responses, a patient in Sub-Saharan Africa could wait upwards of 4 months to initiate treatment after cervical cancer diagnosis, a wait time significantly longer compared to higher income countries (Roy et al., 2022, Noh et al., 2022). Organizations including Go Further are uniquely positioned to expand their scope to streamlining timely access to radiotherapy including brachytherapy.

### 4.3 Radiotherapy needs

Radiotherapy, including EBRT and brachytherapy, is essential in cancer care globally, with chemoradiation as the recommended curative-intent standard of care for locally advanced cervical cancer since 1999 (Green et al., 2005). The typical treatment course includes approximately five weeks of daily EBRT five days per week, along with once weekly concurrent chemotherapy (CRT), with brachytherapy boost performed near or at the end of CRT, with an overall treatment time goal of 8 weeks (Network and Guidelines, 2024). A Lancet Commission on Global Radiotherapy showed RT to be a cost-effective treatment modality (Atun et al., 2015). Despite this, there are many barriers present to RT access, including high upfront costs, electricity requirements, and human resources needs, among others (ref). This limits the availability of this treatment modality in lower resource settings, including Sub-Saharan Africa (Elmore et al., 2021).

In this survey study, over 25 % of respondents reported that their facility lacks a linear accelerator, and almost half of respondents reported their facility lacks a cobalt machine. Over 50 % of respondents indicated having one and two linear accelerator and brachytherapy machines available, respectively. In addition, roughly 20 % of linear accelerators and 50 % of cobalt machines were reported nonfunctional at the time of survey completion, with an estimated average monthly machine downtime greater than 2 weeks. Factors limiting the number of available machines and affecting average monthly downtime create bottlenecks in treatment availability, impacting the total capacity for cervical cancer care in the facility.

With over 100 years since its first use, brachytherapy is a required component of curative-intent cervical cancer treatment, allowing administration of curative doses of radiation to the tumor while avoiding other organ toxicities (Korenaga et al., 2022). Similar to linear accelerator availability, over 25 % of respondents reported that their facility lacks a brachytherapy machine. Of those that do have access to brachytherapy, more than half have access to only one machine. All respondents with brachytherapy access indicated the machine was functioning at the time of survey completion, and 90.9 % reported average monthly downtime of less than 4 days. Brachytherapy does have unique resource requirements, and availability is more concentrated in certain areas of Sub-Saharan Africa including South Africa and Kenya (Bishr and Zaghloul, 2018).

Given these challenges, partnerships with organizations like Go Further could offer neceSub-Saharan Africary funding for interventions that provide new machines and decrease downtime of existing machines, which are essential for future radiotherapy initiatives in Sub-Saharan Africa.

### 4.4 Communication and follow-up needs

Communication between the patient and treatment team is important for a successful treatment course for any type of cancer. While verbal communication was reported as the primary method for explaining a treatment course and side effects to a patient, this method is not without limitations such as increased patient anxiety, unintentional omission of key side effects, and unclear provider communication or patient understanding of treatment timeline (Westendorp et al., 2022, Daskivich et al., 2025). Few facilities reported using more streamlined patient handouts or informational videos, which combat lower health literacy rates among this population by tailoring information to varying literacy levels and languages. By bridging this communication gap and offering additional methods for communicating information, this can facilitate patient understanding and build rapport with their treatment team, contributing to higher quality of life (Berkman et al., 2011). Additionally, culturally-sensitive informational videos can also help to dismantle misconceptions about cervical cancer treatment, making patients more comfortable and informed on their care plan. One study of patients with breast cancer reported that those who received a customizable brochure regarding their treatment were more likely to identify their treatment and participate in shared decision making (Villarreal-Garza et al., 2023). Another study found that patients with only a primary of high school education desired more information about their radiotherapy treatment and preferred this information to be given in pamphlet form (Li et al., 2022). Thus, additional communication methods may improve cancer care in Sub-Saharan Africa. As cervical cancer patients in Sub-Saharan Africa often come from rural areas with lower levels of education, this further supports the growing need for variety in information sharing modalities, such as pamphlets, information campaigns, and visual aids, in the region (Friebel-Klingner et al., 2022).

Regarding patient follow-up after treatment completion, in-person survivorship care appointments are standard among all respondents. However, less than half of respondents reported the use of phone calls as an additional method for follow-up. Given the sparsity of cancer treatment centers in Sub-Saharan Africa, and with patients traveling a median distance of 537 km, follow-up via phone calls or similar form of telehealth is essential for adequate post-treatment care (Patel et al., 2023). Integrating telehealth into the larger system of cancer care in Sub-Saharan Africa not only combats transportation barriers for patients, but also has the potential to reduce the burden on overcrowded cancer care centers. The results of this survey fit into the existing framework of research on communication and follow-up needs in Sub-Saharan Africa, emphasizing the importance of efficient information sharing with patients and additional strategies for follow-up.

### 4.5 Patient barriers to treatment

Previous studies have characterized common barriers to treatment for cervical cancer patients in Sub-Saharan Africa (Friebel-Klingner et al., 2022, Grover et al., 2023). Barriers to timely care can be broadly categorized into (a) patient-related delays in seeking care, (b) treatment-related delays in care, and (c) overall delays representing a combination of these two areas. Patient-related delays in seeking care include factors such as fear of treatment, preference for traditional medicine, and limited knowledge of cervical cancer treatment. Treatment-related delays in seeking care include factors such as travel burden for patients, lack of machine availability, and lack of telehealth infrastructure in the region. In this survey, the most commonly cited barrier to treatment was lack of access to transportation, reported by over 85 % of respondents. A 2022 study from Botswana showed that the median time for women to travel to cervical cancer treatment sites was 2 h (Friebel-Klingner et al., 2022). Another 2023 study published that less than 25 % of patients in Sub-Saharan Africa are within a 2 h travel time of a radiotherapy center (Nadella et al., 2023). An extended travel time for treatment is not only an inconvenience, but also impacts treatment outcomes by increasing delays and worsening cancer outcomes. The same Botswana study found a positive association between a longer travel time and more severe disease stage presentation (Friebel-Klingner et al., 2022). The implications of these delays are considerable. While standard-of-care treatment for locally advanced cervical cancer is completed in 56 days, only 27.6 % of survey respondents reported that >95 % of their patients achieved this benchmark. These results not only call attention to the demand for improved access to transportation, but also for expansion of radiotherapy services to more rural areas in Sub-Saharan Africa.

Other barriers to treatment for cervical cancer patients cited among respondents included lack of knowledge about treatment, lack of familial support, fear of treatment, and social stigma against cervical cancer and HIV. While this survey did not differentiate delays by HIV status or treatment intent, women living with HIV often face additional obstacles such as social stigma against their condition. The combination of these factors create a complex web of barriers patients face in seeking timely treatment. As emphasized previously, these barriers can be addressed by improving communication methods between oncology practitioners and their patients. It has been previously established that patient-centered care with emphasis on shared decision making increases patient understanding of their treatment and quality of life (Elkefi and Asan, 2023). Thus, empowering patients through pamphlets and informational videos during visits can help address these barriers.

More than half (60 %) of respondents cited patient preference for traditional medicine in lieu of chemoradiotherapy as an additional barrier to treatment. While chemoradiotherapy has been established as the standard of care for locally advanced cervical cancer, oncology providers must acknowledge the perspective of their patients in their own cultural context. A systematic review of the use of traditional medicine in Sub-Saharan Africa found that over half of patients using traditional medicine failed to disclose this to healthcare professionals out of fear of receiving subpar care and negative responses from providers (James et al., 2018). This underscores the importance of effective patient information sharing and presents an opportunity for healthcare providers to foster relationships with traditional healers. A 2021 study from northern Uganda also proposed broader acceptance of traditional health practitioners and communication with oncology providers by the national health system to streamline patient care plans (Mwaka et al., 2021).

The results from this survey suggest supplemental avenues to improve access to radiotherapy in Sub-Saharan Africa beyond increasing the number and distribution of radiotherapy machinery in the region. Funding transportation programs, expanding patient education, and supporting traditional healers are all modifiable barriers that programs including Go Further could aim to address.

### 4.6 Limitations and strengths

The primary limitation of this study is the small sample size of 15 oncology providers in Sub-Saharan Africa. This restricts the study’s generalizability, especially to countries outside of the Go Further funding network. The scope of the survey, while covering a wide range of countries, also lacked certain elements. This survey did not cover specific reasons for machine downtime, which could hinder formulation of interventions for reducing downtime in the coming years. Additionally, more detailed radiotherapy information that affects treatment outcomes, including planning techniques and modality of image guidance used for brachytherapy, was not reported, marking areas of expansion for future surveys. Despite its limitations, the common responses of this survey should motivate the development of extended studies investigating these themes in other LMICs beyond South Africa and those funded by Go Further. This survey is focused on provider perspectives on cervical cancer treatment, which lacks patient viewpoints on barriers to treatment and quality of life. However, with provider perspectives as the focal point, this survey captures general trends in radiotherapy access in the represented regions. Another benefit of this study is its multisite approach covering multiple countries with insights into equipment shortages and operational issues. This uncovers new areas of importance for organizations like Go Further to provide funding and strategic planning to ultimately aim to improve cervical cancer outcomes.

### 4.7 Conclusions

This survey of oncology providers in Sub-Saharan Africa highlights the need for investment and deliberate interventions in radiotherapy infrastructure, equipment, and maintenance to streamline treatment for patients with cervical cancer. Commonly reported barriers described in this study, including long wait times for initial appointments, staging, and treatment, the geographic distribution of radiotherapy resources, and barriers in patient communication, represent opportunities for intervention. Investment in long-term initiatives aimed at improving radiotherapy capacity and access in Sub-Saharan Africa can result in massive impact. The WHO predicts that with proper implementation, 14 million women could be saved from death by cervical cancer in 2070, with the cervical cancer incidence rate reducing by 42 % by 2045.^24^ This survey serves as a call to organizations like Go Further, highlighting the urgent need for resource allocation for cervical cancer treatment.

## 5 Presentation

This study was presented as an abstract at the International Gynecologic Cancer Society annual meeting 2024.

## CRediT authorship contribution statement

**Caroline G. Kernell:** Writing – review & editing, Writing – original draft, Methodology, Conceptualization. **Megan K. Kassick:** Writing – review & editing. **Jessica M. George:** Writing – review & editing, Formal analysis. **Chidinma P. Anakwenze:** Writing – review & editing. **Edward L. Trimble:** Writing – review & editing, Supervision, Project administration. **Surbhi Grover:** Writing – review & editing, Supervision, Project administration, Funding acquisition.

## Declaration of competing interest

The authors declare the following financial interests/personal relationships which may be considered as potential competing interests: SG reports consulting fees for Lumonus, Harbinger Health, and Sustainable Dialogue for Peaceful Uses (SDPU) for serving as a scientific advisor. All other authors report no conflict of interest.
